# Tell me how and where you play football and I’ll tell you how much you have to run

**DOI:** 10.5114/biolsport.2022.106155

**Published:** 2021-07-28

**Authors:** Julen Castellano, Ibai Errekagorri, Asier Los Arcos, David Casamichana, Andres Martín-Garcia, Filipe Manuel Clemente, Roberto López-Del Campo, Ricardo Resta, Ibon Echeazarra

**Affiliations:** 1 Society, Sports and Physical Exercise Research Group (GIKAFIT), Department of Physical Education and Sport, University of the Basque Country UPV/EHU, 01007 Vitoria-Gasteiz, Spain; 2 Real Sociedad Football Club, San Sebastian, Spain; 3 Barcelona Football Club, Barcelona, Spain; 4 Escola Superior Desporto e Lazer, Instituto Politécnico de Viana do Castelo, Viana do Castelo, Portugal; 5 Instituto de Telecomunicações, Delegação da Covilhã, Lisboa, Portugal; 6 Department of Competitions and Mediacoach, LaLiga, Madrid, Spain

**Keywords:** Soccer, Team sport, Match analysis, Time-motion, Contextual variables

## Abstract

The aim of this study was to describe the team accumulated physical response per minute considering only the effective playing time (EPT) attending to different contextual and strategic variables in the Spanish Football First Division (*LaLiga Santander*). A sample of 2,959 performances was included in the analysis, considering a 4-year period of analysis (from 2016–17 to 2019–20). The physical variables were: total distance covered with (DTminPOS) and without (DTminNOpos) possession of the ball, and distance covered at > 21 km·h^-1^ with possession (DT21minPOS) and without (DT21minNOpos). Two contextual variables, i.e. Place (Home/Away) and Score (Lost/Draw/Win), and two strategic variables, i.e. level of effective playing time (LevelETP) and level of possession of the ball (LevelPOS), were analysed. The teams ran more without possession of the ball than with possession; nevertheless, the teams that had less possession of the ball had higher values in the distance covered at > 21 km·h^-1^ with possession of the ball and vice versa. Furthermore, the strategic variables also had influence on the physical response (DT and DT21) of the teams,LevelETP and LevelPOS, although with interactive effects: longer playing time, less accumulated distance, and greater possession, greater accumulated distance in the defensive phase, both per min. The findings of this study may offer important practical implications to practitioners in order to assess physical performances of the players in matches, because it is crucial to integrate in the analysis the different contextual and strategic variables where the match has taken place to assess performances of the teams.

## INTRODUCTION

Since computerized tracking systems were implemented in the professional soccer field, many studies have focused their attention on the analysis of physical demands [1]. Nevertheless, several years ago Carling [2] suggested that there is a need for a more pragmatic approach to interpreting the current body of time–motion analysis data. This proposal reveals the difficulty to account for the association between physical match-play response and success in professional soccer, the interpretation of the differences in time-motion analysis data across playing positions, and the use of the time-motion data to identify the presence of fatigue in match-play. In this line, Castellano [3] showed that the physical response (specifically the total distance covered during the match) is not related to the success obtained by the teams at the end of the championship (e.g., accumulated points). It seems that factors other than physical activity per se are more important in achieving success such as the number of shots (but overall, their accuracy), the number of corners, and the number of passes and their efficiency [4, 5]. The inclusion in the model of the many potential confounding factors that can affect physical performance discussed in the academic literature (such as score, place) is necessary [2].

One of the main factors that influences distance covered is the effective playing time (EPT) of the match [6]. Usually, the EPT accounts for a little over 50% of the total match time [6]. Consequently, a time-motion analysis based on EPT (~70% of workload corresponds to this period) can provide more precise information about a player’s physical activity, which may have direct repercussions for the match outcome [6]. Therefore, not considering EPT could lead to a bias when it comes to connecting physical demands with team performance. On the other hand, having or not having the ball in the football is crucial, clearly indicating different phases of the game, attack and defence. The results of some studies that have examined the differences between the two phases of play (attack and defence) show that the distance covered by better teams when they have the ball is greater than that covered by the worst teams [7, 8, 9]. However, better teams run more in ball possession because they have more possession and therefore they have more time to run in this phase of the match. There seems to be increasing evidence that style of play has a clear effect on the physical response of players [10, 11], although the results are inconclusive. Therefore, relativizing the physical response to each minute of ball possession or non-possession could be another interesting strategy for an adequate interpretation of competitive physical response.

The results of the academic literature emphasize the importance of accounting for contextual variables such as opponent level (e.g., high, medium and low), match location (e.g., at home or away) and match status (e.g., winning, drawing or losing), among others, during the assessment of the physical response of soccer performance [1, 9, 12, 13]. These studies showed that soccer players perform significantly less high intensity activity when winning than when losing or drawing [1, 12], suggesting that teams use their maximal physical capacity during the match just when it is essential (pacing effect). In some phases of the match, when losing for example, teams are obligated to try alter the score, and then they might increase the rhythm of the game with the aim of reversing the unfavourable position [12]. With respect to match location (e.g., home or away), no definitive conclusion can be drawn from the previous studies. While Castellano et al. [1] did not find significant differences for distances covered at different speed categories, Lago-Peñas et al. [12] found that differences existed in physical response. This incongruence could be explained by the influence of the interaction with the other contextual variables; probably, as with the previous contextual variable (Score), the probability of the home teams winning the match was greater and so they needed to cover less distance than the rivals. To date, no study has evaluated the effect of contextual variables on distance covered in football considering the effective playing time and possession of the ball from a relative approach (per minute), which has critical importance in the physical performances of the players in team sports. For that reason, the aims of this study were: first, to find out if teams cover more distance in ball possession or not, both in total distance and in high-speed running (> 21 km∙h^-1^), only considering the EPT; second, to determine whether strategic and contextual variables such as the level of EPT, the teams’ level of possession of the ball, the match location (home/away) and the final match score (draw/lose/win) affect the team’s physical response; and third, to assess whether there is a correlation among the physical responses of teams when they have possession of the ball and when they do not. The results of the present study would allow football professionals to assess the physical response in competition as a consequence of the particular competitive scenarios in order to prepare players/teams during the training process.

## MATERIALS AND METHODS

### Experimental approach to the problem

An observational analytic study consisting of a longitudinal 4-year study including all teams from the Spanish Football First Division was performed. Data collection was carried out from the season 2016–17 to 2019–20, using the computerized multi-camera tracking system *TRACAB.*Analysis of variation of total and high-speed running distances per min were performed between independent variables (LevelEPT, LevelPOS, Place, and Score) separately and in interaction.

### Subjects

This study was elaborated using the teams’ performances from the Spanish Football First Division (*LaLiga Santander*) for four seasons (from 2016–17 to 2019–20). Those matches where the information required was not available were excluded (e.g., technical errors). As a result, out of a possible 3,040 teams’ performances (4 seasons * 380 matches * 2 teams’ performances in each match), a total of 2,959 teams’ performances were included in the analysis. Data were obtained from the Spanish Professional Football League, which authorised the use of the variables included in this investigation. In accordance with its ethical guidelines, this investigation does not include information that identifies football players. Data were treated in accordance with the Declaration of Helsinki, and the Ethics Committee on Humans (CEISH) of the University approved their use.

### Physical variables

The present study analysed the physical response considering the total distance and distance > 21 km·h^-1^ covered by teams exclusively in the effective playing time (EPT). Similar to a previous study [10], time in possession of the ball was also considered to analyse the physical demand. In this sense, two different moments of the game, possession and non-possession of the ball, were used to calculate the distance covered by all players of the team in each match. In order to compare the physical demands in matches of different durations of EPT, distances covered were relativized per minute. As a result, four physical variables were obtained: total distance covered in possession (DTminPOS) and without possession (DTminNOPOS) of the ball, and distance covered at > 21 km·h^-1^ in possession (DT21minPOS) and without possession of the ball (DT21minNOPOS).

### Contextual and strategic variables

Four independent variables (contextual and strategic variables) were included in the study: two contextual variables, i.e. match location (Place) and match score (Score), and two strategic variables, i.e. level of effective playing time (LevelEPT) and level of possession (LevelPOS). With respect to the contextual variable match location, and in line with previous studies [14], matches played at home and away were distinguished. With respect to the final outcome or match score [6], it was divided into three levels, e.g., based on whether the team wins, loses or draws the match. Regarding strategic variables, the EPT and POS of the matches were grouped by percentiles in three levels. Each match was classified in one of the three LevelEPT considering the time spent in play in the match: less than 46.4 min (percentile 25%), between 46.5 and 56.1 min (percentiles >25% & <75%), and more than 56.2 min (percentile 75%), being coded as lowEPT, mediumEPT and highEPT, respectively. Also, three LevelPOS levels were established grouping teams’ performances in low, medium and high ball possession, regarding the difference between teams in the time spent in possession of the ball with respect to the rival: less than -7.2 min (percentile 25%), between -7.1 and 7.1 min (percentiles >25% & <75%), and more than 7.2 min (percentile 75%), being coded as lowPOS, mediumPOS and highPOS, respectively. The number of records included is described in Table 1.

**TABLE 1:** Distribution of the records according to the contextual and strategic variables.

Place	Score	LevelPOS	LevelEPT			Total
			lowEPT	mediumEPT	highEPT	
Home	Lost	lowPOS	7	35	16	58
		mediumPOS	45	84	25	154
		highPOS	14	48	18	80

	Draw	lowPOS	9	26	14	49
		mediumPOS	56	67	19	142
		highPOS	15	51	27	93

	Win	lowPOS	30	71	55	156
		mediumPOS	136	206	77	419
		highPOS	37	126	104	267

Away	Lost	lowPOS	41	133	109	283
		mediumPOS	147	218	78	443
		highPOS	34	80	60	174

	Draw	lowPOS	19	55	27	101
		mediumPOS	66	75	21	162
		highPOS	13	29	14	56

	Win	lowPOS	15	54	21	90
		mediumPOS	48	90	25	163
		highPOS	9	41	19	69

	**Total**		741	1,489	729	2,959

Note: lowEPT (>46.4 min), mediumEPT (>46.5 & <56.1 min) and highEPT (>56.1 min) are low, medium and high level of effective playing time (EPT), respectively; and, lowPOS (<-7.2 min), mediumPOS (>-7.1 & <7.1 min) and highPOS (>7.2 min) are low, medium and high level of possession of the ball, respectively.

### Procedures

Time-motion data were obtained by the computerized multi-camera tracking system TRACAB (ChyronHego, New York, USA) and duration of the ball possession was obtained by OPTA Sportsdata (Opta Sports, London, UK), both using Mediacoach software. The reliability of the OPTA system has been previously proved [15] and the reliability of the TRACAB video-tracking system has also been recently tested for physical demand [16, 17], showing a good quality of the data. The generated reports were exported into Microsoft Office Excel (Microsoft Corporation, Washington, USA). A matrix was configured and later analysed using the software JASP version 0.13 (University of Amsterdam, https://jasp-stats.org/, Amsterdam, The Netherlands).

### Statistical analyses

Descriptive statistics data from variables were presented using mean and standard deviation. Tests for normality (Shapiro–Wilk) and equality of variances (Levene’s) were applied. The analysis of variance (ANOVA) for independent samples was used to test for differences in the physical responses (DTmin and DT21min) between independent variables (LevelEPT, LevelPOS, Place, and Score). Also a Pearson correlation analysis was implemented among physical responses: DTminPOS, DTminNOPOS, DT21minPOS and DT21minNOPOS. As proposed by Hopkins [18], the following qualitative correlation descriptors were used: trivial (0–0.09), small (0.1–0.29), moderate (0.3–0.49), large (0.5–0.69), very large (0.7–0.89), nearly perfect (0.9–0.99), and perfect (1). The level of significance was set at p < 0.05.

## RESULTS

The descriptive values (mean and ± standard deviation) of each of the physical variables were as follows: 1434.6 ± 128.7 for DTminPOS, 1565.5 ± 137.1 for DTminNOPOS, 103.7 ± 25.4 for DT21minPOS and 123.0 ± 29.6 for DT21minNOPOS, all in m·min^-1^. Table 2 shows the values of physical response regarding contextual and strategic variables. There were differences (p < 0.05) for DTminPOS and DTminNOPOS in LevelEPT and LevelPOS, and for DT21minPOS and DT21minNOPOS in the four contextual and strategic variables (Place and Score and, LevelEPT and LevelPOS, respectively).

**TABLE 2:** Mean (M) and standard deviation (SD) of the physical responses regarding contextual and strategic variables.

Contextual and strategic variables		Physical responses (m·min^-1^)					
		DTminPOS			DTminNOPOS		
Place		**Home**	**Away**		**Home**	**Away**	
	M	1424.4	1443.7		1578.8	1552.5	
	SD	120.6	124.8		133.8	126.9	

Score		**Lost**	**Draw**	**Win**	**Lost**	**Draw**	**Win**
	M	1443.3	1440.4	1421.9	1554.1	1567.8	1574.9
	SD	118.6	120.1	128.3	131.7	127.8	130.9

LevelEPT		**lowEPT**	**mediumEPT**	**highEPT**	**lowEPT**	**mediumEPT**	**highEPT**
	M	1484.2^**gh**^	1434.8^**h**^	1382.4	1605.5^**gh**^	1568.3^**h**^	1517.4
	SD	114.9	109.1	136.7	126.6	121.4	138.7

LevelPOS		**lowPOS**	**mediumPOS**	**highPOS**	**lowPOS**	**mediumPOS**	**highPOS**
	M	1514.2^**jk**^	1439.9^**k**^	1343.3	1484.6	1568.4^**i**^	1638.6^**ij**^
	SD	123.2	100.4	103.6	115.0	111.3	136.9

		DT21minPOS			DT21minNOPOS		

Place		**Home**	**Away**		**Home**	**Away**	
	M	105.1^**b**^	102.4		125.69^**b**^	120.6	
	SD	25.8	25.0		29.2	29.8	

Score		**Lost**	**Draw**	**Win**	**Lost**	**Draw**	**Win**
	M	99.1	105.7^**c**^	107.3^**cd**^	123.6^**e**^	124.4	121.8
	SD	23.6	27.2	25.6	29.7	30.5	29.1

LevelEPT		**lowEPT**	**mediumEPT**	**highEPT**	**lowEPT**	**mediumEPT**	**highEPT**
	M	114.2^**gh**^	102.7^**h**^	94.8	135.7^**gh**^	122.2^**h**^	111.8
	SD	25.3	23.1	25.8	28.7	26.9	30.5

LevelPOS		**lowPOS**	**mediumPOS**	**highPOS**	**lowPOS**	**mediumPOS**	**highPOS**
	M	117.5^**jk**^	105.1^**k**^	87.2	102.8	124.9^**i**^	139.3^**ij**^
	SD	25.7	23.0	19.4	23.7	26.5	28.9

Note: Distance covered in possession (DTminPOS) and in no possession (DTminNOPOS) of the ball, and distance covered at > 21 km·h^-1^ in possession (DT21minPOS) and no possession of the ball (DT21minNOPOS). LowEPT, mediumEPT and highEPT are low, medium and high level of effective playing time (EPT), respectively; and, lowPOS, mediumPOS and highPOS are low, medium and high level of possession of the ball, respectively.

a is > home, b is > away, c is > loss, d is > draw, e is > win, f is > LowEPT, g is > mediumEPT, h is > highEPT, i is > lowPOS, j is > mediumPOS and k is > highPOS (p < 0.05).

**FIG. 1:**
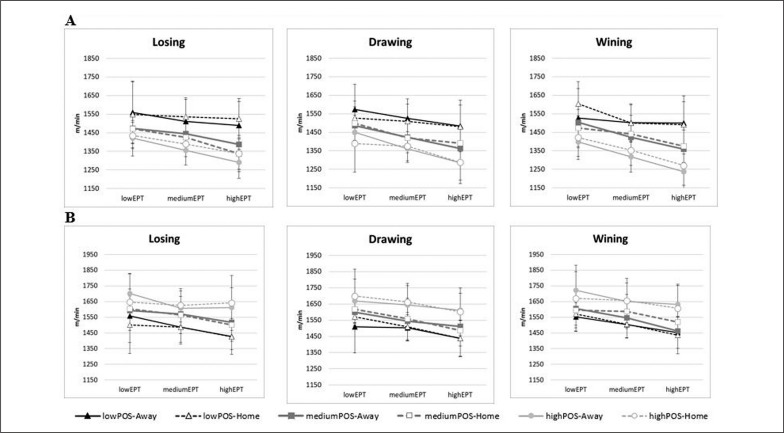
Total distance per minute (m·min^-1^) in possession (A = DTminPOS) and without possession (B = DTminNOPOS) covered by teams regarding four contextual and strategic variables: Score (Draw, Loss and Win), Place (Home and Away), LevelPOS (lowPOS, mediumPOS, and highPOS) and LevelEPT (lowEPT, mediumEPT and highEPT). lowEPT (> 46.4 min), mediumEPT (> 46.5 & < 56.1 min) and highEPT (> 56.1 min) are low, medium and high level of effective playing time (EPT), respectively; and, lowPOS (< -7.2 min), mediumPOS (> -7.1 & < 7.1 min) and highPOS (> 7.1 min) are low, medium and high level of possession of the ball, respectively.

Figure 1 shows the comparison between the variables of total distance covered with (DTminPOS) and without (DTminNOPOS) possession of the ball per minute, considering the contextual and strategic variables (Score, Place, LevelEPT and LevelPOS).

**FIG. 2:**
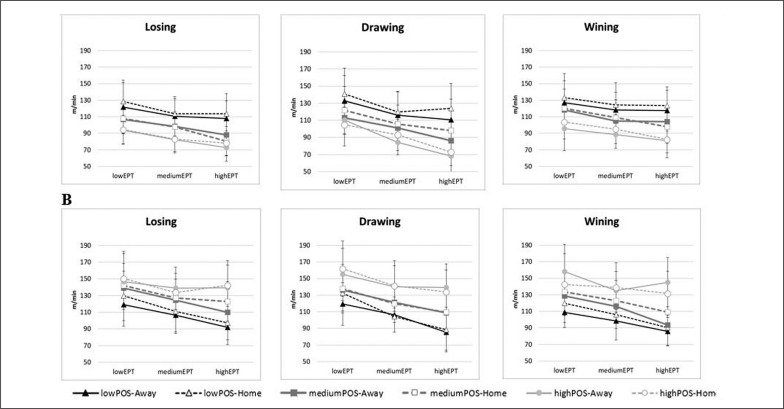
Total distance per minute (m·min^-1^) at > 21 km·h^-1^ in possession (A = DT21minPOS) and without possession (B = DT21minNOPOS) covered by teams regarding four contextual and strategic variables: Score (Draw, Loss and Win), Place (Home and Away), LevelPOS (lowPOS, mediumPOS, and highPOS) and LevelEPT (lowEPT, mediumEPT and highEPT). lowEPT (> 46.4 min), mediumEPT (> 46.5 & < 56.1 min) and highEPT (> 56.1 min) are low, medium and high level of effective playing time (EPT), respectively; and, lowPOS (< -7.2 min), mediumPOS (> -7.1 & < 7.1 min) and highPOS (> 7.1 min) are low, medium and high level of possession of the ball, respectively.

Figure 2 shows the comparison between the variables of distance covered at > 21 km·h^-1^ with (DT21minPOS) and without (DT21minNOPOS) possession of the ball per minute, taking into account the contextual and strategic variables (LevelEPT, LevelPOS, Place and Score).

**FIG. 3:**
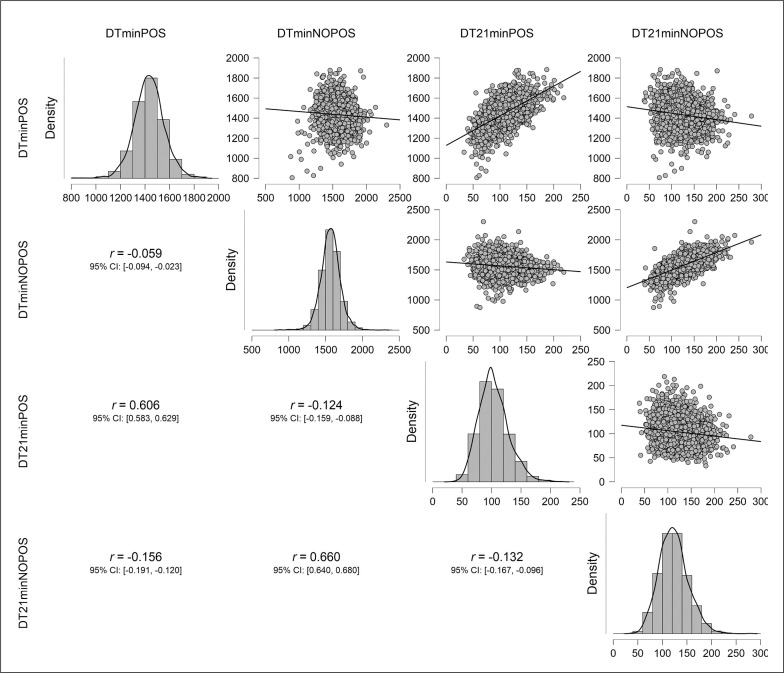
Pearson correlation values among physical demands. Distance covered in possession (DTminPOS) and without possession (DTminNOPOS) of the ball, and distance covered at > 21 km·h^-1^ in possession (DT21minPOS) and without possession of the ball (DT21minNOPOS).

Pearson correlations between physical responses are presented in Figure 3. A large positive correlation (p < 0.01) was found between DTminPOS and DT21minPOS (r = 0.61) and between DTminNOPOS and DT21minNOPOS (r = 0.66), but not between possession and non-possession of the ball.

## DISCUSSION

The study aim was to describe the physical response to four contextual and strategic variables in matches played during four seasons (from 2016–17 to 2019–20) in LaLiga Santander. As far as the authors know, this is the first study that analyses physical teams’ performances in LaLiga Santander by only taking into account the EPT and distinguishing the two main phases of the game, having or not having the ball, and normalized to meters per minute (m·min^-1^). The results suggest several interesting particularities in the physical responses of the teams during matches: firstly, teams run more in the non-possession phase than in the ball-possession one; secondly, regarding the four strategic and contextual variables, mainly the first, EPT and amount of ball possession (POS in %) had a great influence on the running response. Finally, all four variables had an effect on the physical responses of the players: there was greater distance accumulated per minute at home, the score being drawn, with low POS and low EPT.

Some previous studies [7, 8, 19] have tried to connect ball possession with physical demand, concluding that better teams cover greater distance with ball possession. But support of this conclusion is that better teams run more because they have more time to run in the possession phase of a match. Contrary to what could be interpreted from previous studies, a study recently described the influence of ball possession on the running demand of players [20]. This study concluded that teams with a high percentage of possession seem to require a lower conditional response with respect to teams with less ball possession. The results of the present study complement this contribution, since it can be verified that, in general terms, each minute of possession requires a lower physical response, both in total amount (DT) and accumulated at > 21 km·h^-1^.

However, this general statement that the defensive or non-ball-possession phase is more physically demanding is affected by certain contextual variables, such as Score and Place, but especially by strategic variables such as the time of possession and the time in which the ball is in play during a match [1]. The study of physical demand during EPT is not new [1], although it has recently received some attention again [21, 22]. As might be expected, in competition most of the distance is covered players at the moments when the ball is in play [1], accumulating a higher percentage of the total as the speed ranges increase (e.g., > 21 km·h^-1^). Contrary to what might be expected, higher LevelEPT values, the highest effective playing time, had an opposite effect on the relative distance covered both in ball possession and without possession of the ball. In reality, teams ran a shorter distance in both DT and DT21 when the EPT was longer in the match (lowEPT > mediumEPT > highEPT). It should be noted that the pattern is repeated for both DT and DT21 with and without ball possession, and considering the variables of LevelPOS, Score and Place.

Regarding the second strategic variable, possession of the ball, the results of the present study contradict those reported by Yi et al. [11], which showed that possession-play characterized teams achieved higher values of distance in sprints and high-intensity running. Probably, methodological criteria used to group teams with a more direct or indirect style of play (possession-play style) were the main reason for this difference. A relevant aspect of the results is that the percentage of possession of the teams also had an influence on the distance covered over 21 km h^-1^: the DT21minPOS values were higher when the team had less ball possession than opponent team (lowPOS); in contrast, high values in DT21minNOPOS occurred in those teams that dominated ball possession (highPOS). These results agree with those provided in the study of Castellano & Echeazarra [10], where they found that teams with a more direct style of play, and therefore with less possession, had a greater conditional response, especially in the accumulated run in the offensive phase, contrary to what is demanded by a way of playing clearly based on possession. Everything indicates that teams with direct playing styles and fast movements with the ball require a particular physical condition that allows players to respond to this demand [23].

It is not new that contextual variables affect team games [24] and, therefore, the physical response of players [1, 2, 12, 13]. In the present study, the contextual variables Score and Place influenced the distance covered at > 21 km·h^-1^, but not the total distance accumulated by the teams, with and without possession of the ball. These results partially agree with the study of Lago-Peñas and colleagues [12], which concluded that the home teams covered a greater distance than visiting teams at low intensity (< 14.1 km·h^-1^), but no differences were observed at medium, sub-maximal or maximal intensities. Nevertheless, regarding the distance covered at high speed (> 21 km·h^-1^) both of them, Score and Place, were found to have an influence on physical demands: more distance in the variables DT21minPOS and DT21minNOPOS for home teams and when teams had drawn. The difference between the studies may be due to the fact that the physical performance evaluated in the present study is limited exclusively to effective playing time.

Regarding the correlation between the variables DT and DT21, two interesting aspects can be highlighted. On the one hand, the teams that accumulated the greatest total distance also covered a greater distance at > 21 km h^-1^ within the same offensive or defensive phase of the match. On the other hand, there was no correlation between the relative distance with possession of the ball and the relative distance without either in the total distance or in the distance at >21 km·h^-1^ (Figure 3). From these results, it could be interpreted that teams ‘choose’ their style of play depending on the phase of the match; with or without ball possession, players have a more demanding conditional response, not being able to address or maintain a highly demanding conditional response in both phases simultaneously. This corroborates the need to assess performance in competition considering the style of play that the team has, both in the offensive phase (ball-possession period), with a more direct, counterattack or combined style, and in the non-possession or defensive phase, using a more pressing or deep-defence playing style [25]. The strong correlation between DT and DT21 provides a better understanding of the style of play and physical response relationship and therefore gives better insight into teams’ performances.

This research study is not without limitations. First, the possible technical errors inherent to the technology with which the records have been made must be considered [16]. Secondly, with regard to the contextual variable Score, the fact that the match has been catalogued based on the final result makes it very likely that a different way of scoring (e.g., match status) that could have been used during these matches was overlooked and could have changed the three types of established markers. Third, the use of a strategy other than the one used with respect to percentiles to segment and classify the teams in the levels of ball possession or effective playing time of the match could have affected the results obtained in the game in the present study.

## CONCLUSIONS

The main conclusions of the study were as follows: 1) teams ran more per minute when players did not have the ball than when they did; 2) the teams that accumulated greater total distance per minute also covered greater distance at >21 km·h^-1^ per minute; 3) the teams that had less possession of the ball had higher values in the distance covered at >21 km·h^-1^ per minute with ball possession, whereas those that had greater possession of the ball accumulated greater distance covered at >21 km·h^-1^ per minute in the non-possession phase; 4) the contextual variables Score and Place affected the distance covered at >21 km·h^-1^ but not the total distance accumulated per minute by the teams, running greater distance in matches that finished in a draw and when played at home; 5) the strategic variables had more influence on the teams’ physical response (DT and DT21), LevelETP and LevelPOS, although with interactive effects with contextual variables: longer EPT, less accumulated distance per minute, and greater possession, greater accumulated distance in the non-possession phase; and 6) the distance accumulated per minute by the teams in ball possession does not correlate with the distance accumulated in the non-possession phase and vice versa.The practical application derived from the results of this study concerns, above all, sensitizing practitioners when they intend to evaluate the physical performance of players in matches. It is crucial to incorporate in this assessment the different contextual variables where the match has developed, as well as the particular strategic variables that the teams have proposed.
